# Personalised fluid resuscitation in the ICU: still a fluid concept?

**DOI:** 10.1186/s13054-017-1909-5

**Published:** 2017-12-28

**Authors:** Frank van Haren

**Affiliations:** 1 University of Canberra, Canberra, Australia; 2 Australian National University, Canberra, Australia; 3 Intensive Care Unit, Canberra Hospital, Canberra, Australia

**Keywords:** Fluid, Fluid bolus, Intensive care, Sepsis, Resuscitation, Shock, Personalised

## Abstract

The administration of intravenous fluid to critically ill patients is one of the most common, but also one of the most fiercely debated, interventions in intensive care medicine. Even though many thousands of patients have been enrolled in large trials of alternative fluid strategies, consensus remains elusive and practice is widely variable. Critically ill patients are significantly heterogeneous, making a one size fits all approach unlikely to be successful.

New data from basic, animal, and clinical research suggest that fluid resuscitation could be associated with significant harm. There are several important limitations and concerns regarding fluid bolus therapy as it is currently being used in clinical practice. These include, but are not limited to: the lack of an agreed definition; limited and short-lived physiological effects; no evidence of an effect on relevant patient outcomes; and the potential to contribute to fluid overload, specifically when fluid responsiveness is not assessed and when targets and safety limits are not used.

Fluid administration in critically ill patients requires clinicians to integrate abnormal physiological parameters into a clinical decision-making model that also incorporates the likely diagnosis and the likely risk or benefit in the specific patient’s context. Personalised fluid resuscitation requires careful attention to the mnemonic CIT TAIT: context, indication, targets, timing, amount of fluid, infusion strategy, and type of fluid.

The research agenda should focus on experimental and clinical studies to: improve our understanding of the physiological effects of fluid infusion, e.g. on the glycocalyx; evaluate new types of fluids; evaluate novel fluid minimisation protocols; study the effects of a no-fluid strategy for selected patients and scenarios; and compare fluid therapy with other interventions. The adaptive platform trial design may provide us with the tools to evaluate these types of interventions in the intrinsically heterogeneous intensive care unit population, accounting for the explicit assumption that treatment effects may be heterogeneous.

## Background

Although there is no universally accepted definition, personalised medicine has been described as “a medical model using characterization of individuals’ phenotypes and genotypes (e.g. molecular profiling, medical imaging, lifestyle data) for tailoring the right therapeutic strategy for the right person at the right time, and/or to determine the predisposition to disease and/or to deliver timely and targeted prevention”. This definition was used by EU Health Ministers in their Council conclusions on personalised medicine for patients, published in 2015 [1].

Because of the way intensive care units (ICUs) have developed over the years, one could argue that they perhaps offer the ultimate environment for real-time personalised medicine. Modern ICUs provide a plethora of readily available patient-specific data, such as point of care blood tests, invasive and non-invasive haemodynamic and respiratory measurements, bedside ultrasound, and many other measures and variables, to help guide therapy. In addition, intensive care patients are significantly heterogeneous, underlining the need for personalised medicine principles.

The administration of intravenous fluids is one of the most common interventions in the intensive care environment. There is on-going discussion regarding the benefits and risks of infusion therapy. While the application of fluid during shock may lead to circulatory stabilisation and can therefore be potentially lifesaving, fluid overload is associated with oedema development and worse outcomes [2]. Interestingly, despite the worldwide use of various infusion solutions, no concrete evidence has been provided that infusion therapy per se leads to a lower mortality among seriously ill patients. In the case of sepsis, the bench-to-bedside evidence supporting fluid resuscitation as a treatment remains remarkably weak and highly conflicting. Our current practice seems mainly to be based on historical beliefs and an incomplete or incorrect understanding of the pathophysiology of sepsis [3].

The paucity of evidence in this area of intensive care medicine may be related to the complexity of treatment in an intrinsically heterogeneous group of critically ill patients, as well as to considerable differences that exist in the clinical use of infusion therapy. Patient heterogeneity, even within groups of defined illnesses such as sepsis or acute respiratory distress syndrome (ARDS), remains one of the main challenges when it comes to developing robust evidence that therapeutic interventions such as fluid administration improve patient-centred outcomes, e.g. mortality and long-term function. The results of several large fluid studies have provided clinicians with some useful answers but also with many more questions and, as a result, a significant level of debate and controversy remains. These large randomised controlled trials, although considered the highest level of evidence, often fail to take into account patients’ heterogeneity, and consequently show negative results [4].

Applying personalised medicine principles to fluid administration in critically ill patients requires careful attention to: context, indication, targets, timing, amount of fluid, infusion strategy, and type of fluid.

## Context, indication, and targets

It is important to differentiate between fluid substitution and volume substitution in intensive care patients. Different indications warrant different strategies and fluid choices, a distinction that has not always been appreciated sufficiently in the design of fluid studies. In addition, and again touching on patient heterogeneity, an intervention may be beneficial in one group of patients and harmful in another. This was elegantly demonstrated in a recent systematic review and meta-analysis of blood product transfusion strategy in the perioperative and acute care setting. In this study, the authors used a context-specific approach, based on patient characteristics and clinical settings, to estimate the risk or benefit of a restrictive transfusion strategy as compared with a liberal transfusion strategy. They found that a restrictive transfusion strategy was associated with an increased risk of composite cardiovascular events and death in patients with cardiovascular disease undergoing cardiac or vascular procedures, as well as in elderly patients undergoing orthopaedic surgery, but not in a mixed group of critically ill patients [5].

As could be expected, the clinician’s diagnosis or working hypothesis of which underlying illness or process leads to abnormal physiology plays an important role in the decision-making process regarding fluid resuscitation. For example, in one Australian study, recognition and physician documentation of sepsis syndrome in patients presenting to the emergency department resulted in administration of significantly more volume of fluid [6]. This may seem straightforward, but unfortunately that is not the case. Diagnosing sepsis requires interpreting non-specific signs and can therefore be subjective and variable. In a recent survey of intensivists in the USA, researchers presented case vignettes of patients with suspected or confirmed infection and organ dysfunction. They found that the overall inter-rater agreement in sepsis diagnosis was poor, despite the fact that cases were overall reported as realistic [7]. The importance of clinician recognition has also been shown in other intensive care studies. In the recently published Large observational study to UNderstand the Global impact of Severe Acute respiratory FailurE (LUNG SAFE) study, clinician recognition of ARDS had a significant effect on treatment. Clinicians who diagnosed ARDS were, for example, more likely to apply higher positive end-expiratory pressure (PEEP) levels and to use adjunctive measures, such as proning the patient and using neuromuscular blockade, than clinicians who did not diagnose ARDS [8].

Abnormal physiology is often perceived to reflect a requirement for fluid resuscitation. A significant proportion of the evidence we generate in studies and translate into everyday practice is based on targeting intermediate end-points, such as improvement or correction of abnormal physiology. However, this approach may or may not result in improvement of relevant patient-centred outcomes. An excellent example of this dilemma can be found in studies in which tonometry-guided treatment was used in an attempt to improve outcomes [9]. Gastrointestinal mucosal hypoperfusion, resulting in an increased gastric to arterial difference in partial CO_2_ pressure as measured by gastric tonometry, has been shown to be associated with worse patient outcomes [10]. However, interventions to target this parameter, such as fluid resuscitation or the administration of different types of inotropic and vasoactive medications, have consistently failed to improve patient outcomes [9]. Another example is the use of resuscitation protocols to achieve and maintain urine output above a pre-defined threshold to prevent acute renal failure. In a recent systematic review and meta-analysis, targeting oliguria reversal in goal-directed haemodynamic management did not reduce renal dysfunction in peri-operative and critically ill patients [11]. There are many more examples of this disconnect between the effect of a therapeutic intervention on physiological parameters and the effect on outcome. Clinicians should be mindful of the potential of harming patients by overly aggressive attempts to correct abnormal numbers, and remember lessons well learned over the last few decades.

## Timing, amount of fluid, and infusion strategy

Resuscitation of patients in shock needs to be timely and adequate. The Surviving Sepsis Guidelines suggest that a fluid bolus of 30 ml/kg crystalloid be administered to septic patients with hypotension or elevated lactate levels within 3 h of the time of presentation [12]. Fluid bolus therapy (FBT) is perhaps the most common way intravenous fluids are administered in the ICU. Fluid boluses are usually administered to correct abnormal physiologic parameters, including but not limited to hypotension, tachycardia, oliguria, and increased lactate levels. Despite its widespread use, there are several important limitations and concerns regarding FBT in the acute care setting.

First, there is no universally accepted definition of what a fluid bolus is and how it should be administered, and what physiological effects clinicians expect as a result of a fluid bolus. This was illustrated in a recent survey amongst acute care physicians in Australia and New Zealand [13]. This study showed that FBT is a poorly defined intervention with considerable variability in preferred fluid choice, volume given, and speed of delivery. In addition, intensive care and emergency medicine specialists showed a wide variation in their expected physiological responses to FBT. Similar wide variability between individuals and countries was found when the researchers expanded their study to 3138 practitioners from 30 countries [14]. Another global inception cohort study, the “fluid challenges in intensive care” (FENICE) study, reached the same conclusions: the current practice and evaluation of FBT in critically ill patients are highly variable [15].

Second, the physiological effects of FBT in critically ill patients have not been well studied. This was highlighted in a systematic review of contemporary data of physiological changes after FBT in sepsis [16]. No randomised controlled trials compared FBT with alternative interventions, such as vasopressors. Although 17 studies described the temporal course of physiological changes after FBT in 31 patient groups, only three studies described the physiological changes at 60 min, and only one study beyond this point [17]. No studies related the physiological changes after FBT with clinically relevant outcomes. The authors concluded that there is a clear need for at least obtaining randomised controlled evidence for the physiological effects of FBT beyond the period immediately after its administration [16]. In addition, the most effective rate of FBT is unknown. The rate at which a fluid bolus is administered appears to influence haemodynamic variables. In a randomised cross-over pilot study in a healthy volunteer model of compensated haemorrhagic shock, participants were randomised to receiving 20 mL/kg of crystalloid over 10 min (fast) or 30 min (slow). During fast fluid resuscitation, the blood pressure was higher, but the cardiac index paradoxically decreased in most participants during the resuscitation phase; a finding not observed in the slow group [18].

Third, the haemodynamic response to a fluid bolus is usually small and short-lived, and the clinical relevance of this physiological effect is uncertain. In the previously mentioned systematic review of contemporary data of physiological changes after FBT, the mean arterial pressure (MAP) increased on average by 7.8 mmHg immediately after a fluid bolus, and returned close to baseline after 1 h, with no increase in urine output [16]. These results were confirmed in a well-conducted prospective observational study, in which the duration of hemodynamic effects of crystalloids was assessed in patients with circulatory shock after their initial resuscitation [19]. In this study, patients had to be on vasopressors for at least 6 h, and received 500 ml of crystalloids, infused over 30 min. Responders to the fluid bolus were patients in which the cardiac index increased > 15% immediately after the fluid bolus, as measured by pulmonary artery catheters. The duration of the volume effect was found to be short in all patients, as well as in the sub-group of fluid responders. Cardiac index, blood pressure, and cardiac filling pressures significantly decreased 30 min after the fluid bolus, and returned to baseline levels 60 min after the fluid bolus. These results suggest that volume expansion with crystalloids in patients with circulatory shock after the initial resuscitation has limited success, even in patients who are fluid responsive [19]. In another pragmatic clinical observational study of post-resuscitation FBT in septic patients in the ICU, fluid boluses also had very limited success. In addition, they were potentially harmful by contributing to an overall cumulative positive fluid balance, which was in turn associated with worsening sequential organ failure assessment (SOFA) and lung injury scores [20]. In a retrospective analysis of the ARDS Network Fluid and Catheter Treatment Trial, hemodynamic responses were investigated in a convenience sample of 127 patients. In this study, critically ill patients were given protocol-based crystalloid or albumin boluses for shock, low urine output, or low pulmonary artery occlusion pressure (PAOP). There were significant increases in mean central venous pressure and mean PAOP following fluid boluses. However, there were no significant changes in urine output, and there were clinically small changes in heart rate, MAP, and cardiac index. [21] These observations of clinically small and short-lived haemodynamic responses to FBT are not limited to patients who have already been fluid resuscitated. In shocked patients in the emergency department, the median increase in MAP was only 3 mmHg 1 h after FBT, without an effect on the heart rate [22]. Early goal-directed treatment (EGDT) of septic shock involves protocolised processes of care that usually incorporate more invasive monitoring and the administration of more intravenous fluids, blood transfusions, vasopressors, and inotropes compared with standard care. In several recent large trials, patients assigned to EGDT received significantly more fluids than patients receiving standard care. EGDT consistently failed to show an improvement in mortality for patients with septic shock, but was associated with more ICU admissions and increased utilisation of ICU resources [23]. These findings do not support the systematic use of EGDT, of which more aggressive fluid resuscitation is a component, in the management of patients with septic shock.

Finally, FBT carries a significant potential for harm, especially if used indiscriminately. Rather alarmingly, clinicians do not appear to be particularly good at determining whether a patient will benefit from the administration of a fluid bolus, especially when basing this decision on clinical examination and static haemodynamic indices such as central venous pressure. In studies summarised in a review by Michard and Teboul in 2002 [24], around 50% of patients who received a fluid bolus based on clinical signs and static haemodynamic measurements such as central venous pressure turned out to be not fluid responsive. In other words, the decision-making process to administer a fluid bolus was not much better than tossing a coin. Another important conclusion based on that observation is that we may be causing harm in about 50% of our patients who are given a fluid bolus in error. Fluid resuscitation therefore should ideally be based on dynamic indices of fluid responsiveness, such as stroke volume or pulse pressure variation, but the presence of arrhythmias and spontaneous breathing activity and the use of lung protective ventilation may preclude these indices from being used [25]. The easy-to-perform passive leg raising test has been well validated in situations where these dynamic indices cannot be used reliably in the intensive care environment [26]. Bedside echocardiography and the recently described end-expiratory occlusion test are other means to identify preload responsiveness. The dynamic tests are complementary, and clinicians should choose between them based on the status of the patient and the cardiac output monitoring technique. [27] Unfortunately, this approach does not seem to be routinely practiced in the real world. Clinicians do not widely use measures of fluid responsiveness in their usual practice, as evidenced by the earlier referenced observational FENICE study [15]. Not only was prediction of fluid responsiveness not used routinely, safety limits for FBT were also rarely used. Even more concerning was the observation in this study that there was no statistically significant difference in the proportion of patients who received further fluids after the previous fluid bolus between those with a positive, with an uncertain, or with a negatively judged response to fluids. In other words, patients who were proven to be not fluid responsive continued to receive the same amount of subsequent fluid boluses as did fluid responsive patients [15]. This practice undoubtedly increases the risk of fluid overload in critically ill patients. As already mentioned, FBT has been shown to independently contribute to a cumulative positive fluid balance, with associated organ failure [20]. An increasing number of studies link fluid overload in septic patients to worse outcomes [28–31]. The ARDS Network Fluids and Catheters Treatment Trial showed that a conservative fluid strategy may improve patient outcomes. In this study, 1000 patients with acute lung injury were randomised into a conservative and a liberal strategy of fluid management using explicit protocols, which were applied for 7 days. Patients in the conservative strategy arm showed significantly improved lung function and shorter duration of mechanical ventilation and intensive care without increasing non-pulmonary organ failures [32].

To date, the only randomised controlled trial of fluid resuscitation in sepsis is the FEAST trial (Mortality after Fluid Bolus in African Children with Severe Infection) [33]. The investigators randomised 3141 (of a planned 3600) children with severe sepsis to receive FBT with either 40 mL/kg 0.9% saline or 4% albumin or no-volume resuscitation. The trial was stopped early for harm, demonstrating a 40% increase in mortality in both the FBT arms compared to the no-volume resuscitation. Much has been made with regards to the correct interpretation of these findings [34–36]. It has been suggested that the findings are specific to the unique population with a high incidence of malaria (57%), severe anaemia < 5 g/dL (32%), and acidosis (base deficit > 8 mmol/L, 51%) with saline and albumin causing disease-specific deterioration and worsening of both anaemia and acidosis [34, 35]. However, the published sub-group analysis does not support these conclusions, with similar point estimates for harm independent of prior malaria, baseline haemoglobin, and base deficit [33].

## Type of fluid

Currently, there is no evidence from randomised controlled trials that resuscitation with colloids, instead of crystalloids, reduces the risk of death in critically ill patients [37–39]. Interestingly, significant regional variation exists regarding the use of resuscitation fluids in critically ill patients [40]. Following the publication of several large fluid trials, fluid resuscitation practices appear to have changed globally. For example, in an observational study in Australia and New Zealand, the administration of resuscitation fluids in patients admitted to adult intensive care units was recorded for a 24-h period at six time points between 2007 and 2013 [41]. Over the 6-year period, crystalloid use had increased, primarily due to an increase in the use of balanced salt solutions, and the overall use of colloids had decreased, primarily due to a decrease in the use of gelatins.

Balanced fluids are crystalloid and colloid solutions with a more physiologically balanced electrolyte formulation, such as Hartmann's solution, PlasmaLyte, and Hextend. The use of these fluids for volume resuscitation can potentially prevent the development of hyperchloraemic acidosis, an electrolyte abnormality often encountered with the use of “normal” saline even after relatively low volume application [42–44]. In addition, the use of 0.9% saline has been associated with potential adverse renal effects, possibly mediated by its effects on renal blood flow. For example, in a randomised controlled, double-blind cross-over study on the effects of 2-L infusions of 0.9% saline and PlasmaLyte 148 in healthy volunteers, intravenous infusion of 0.9% saline resulted in reductions in renal blood flow velocity and renal cortical tissue perfusion [45]. A similar difference was seen in a subsequent study, comparing a balanced starch solution with starch in 0.9% saline, showing an increase in renal cortical tissue perfusion in the balanced group compared with the saline group [46]. In experimental sepsis models, balanced fluids are associated with a better short-term survival [47]. In a prospective, open-label, sequential period pilot study in 1533 critically ill patients, implementation of a chloride-restrictive strategy in a tertiary ICU was associated with a significant decrease in the incidence of acute kidney injury and the use of renal replacement therapy [48]. However, in the largest prospective randomised study to date, no signal towards harm was found regarding the use of 0.9% saline in ICU patients compared with PlasmaLyte 148. In this double-blind, cluster randomised, double cross-over trial, the effects of saline versus PlasmaLyte on renal complications in patients admitted to the ICU were compared. No differences between the two groups were found in mortality, development of acute kidney injury, and use of renal replacement therapy [49]. Preparations are underway for the large PlasmaLyte versus Saline (PLUS) trial (NCT02721654) to test the hypothesis that 90-day all-cause mortality will be lower in patients assigned to receive PlasmaLyte for intravenous volume resuscitation and subsequent crystalloid therapy in the ICU compared to those assigned to receive 0.9% saline. Critics of this trial argue that: the design does not take into account patient heterogeneity, and assumes that all patients will be either improved or harmed by one strategy compared with another; the intervention deviates from normal clinical practice in which the choice of fluid usually would be guided by baseline factors and elements arising during therapy such as the results of electrolyte measurements; the outcomes of the trial are predictable because of the trial design; and education of clinicians is more likely to improve patient outcomes [50].

## Future directions

Going forward, we need to focus on several important issues.

First, we need to educate clinicians about the risks of fluid loading patients who are not fluid responsive. The potential of harm caused by fluid bolus therapy should more clearly feature in guidelines. Implementation of a physiologic, haemodynamically guided conservative approach to fluid therapy in patients with sepsis would possibly reduce the morbidity and improve the outcome [51]. The safety, feasibility, and efficacy of targeted fluid minimisation strategies using protocol-guided assessments of fluid responsiveness holds promise, but needs to be further investigated [52].

Second, we urgently need to go back to the drawing board to design rigorous research to re-examine fluid therapy. The effects of fluid infusion on the immune system, on endothelial function, and on the integrity of the glycocalyx remain poorly understood. Degradation of the glycocalyx on the vascular luminal cell membrane has been identified to be an early step in septic vascular endothelial cell disorder [53]. Fluid therapy has the potential to further damage the glycocalyx, especially when rapid infusions are used and when fluid infusion results in hypervolaemia [54–56]. In addition to more basic science-type research, we also require experimental studies that accurately reflect the presentation of human septic shock and clinical studies testing either lower volumes of fluid resuscitation or supportive care without fluid resuscitation. Alternatives to fluid bolus therapy for the treatment of shock, such as the early use of vasoactive drugs, need to be further assessed in prospective randomised studies [57].

Third, the concept of small-volume resuscitation using hypertonic fluids in sepsis deserves additional investigation. Hypertonic resuscitation may provide effective and rapid intravascular volume resuscitation. In addition, some preliminary data suggest that hypertonic fluid administration in sepsis may have beneficial effects on the global circulation and the cardiac function that exceed simple intravascular volume expansion [17]. Hypertonic resuscitation may exert specific effects on inflammatory pathways and endothelial function that may be beneficial in patients with septic shock and acute lung injury [58]. Whether these observations translate into improved clinical outcomes has not yet been established, and much more work is required before this experimental approach is to be implemented into clinical practice [59].

Finally, we should embrace new fluid trial designs that are capable of evaluating efficacy of multiple interventions in a heterogeneous ICU population, explicitly assuming treatment effects may be heterogeneous [60]. Adaptive platform trials are clinical trials with a single master protocol in which multiple treatments are evaluated simultaneously, with added flexibility to drop treatments for futility, implement treatments that are superior, and adding new potential treatments to the trial to be tested. A simulation study comparing platform study designs with traditional two-arm designs found that platform trials can find beneficial treatments with fewer patients, fewer patient failures, less time, and with greater probability of success than a traditional two-arm strategy [61]. Platform trials may provide us with the tools to evaluate different components of fluid therapy in critically ill patients, and with the answers we need to personalise treatment.

## Conclusions

Fluid resuscitation has long been one of the cornerstones of intensive care treatment, albeit with a limited evidence base in terms of its effects on outcome. An increasing body of literature suggests that fluid bolus therapy may contribute to fluid overload and cause harm, partly because clinicians do not routinely test for fluid responsiveness and rarely apply safety limits. The effects of fluid boluses on physiological parameters are not well studied, and seem small and short-lived at best.

Personalised fluid administration in critically ill patients requires clinicians to integrate abnormal physiological parameters into a clinical decision-making model that also incorporates the likely diagnosis and the likely risk or benefit in the specific patient’s context. Personalised fluid resuscitation therefore requires careful attention to the mnemonic CIT TAIT: context, indication, targets, timing, amount of fluid, infusion strategy, and type of fluid.

The research agenda should focus on basic research to improve our understanding of the physiological effects of fluid infusion, e.g. on the glycocalyx, and experimental and clinical studies to evaluate novel fluid minimisation protocols including no fluid strategies, and studies comparing fluid therapy with other interventions. The platform trial design may provide us with the tools to evaluate these types of interventions in the intrinsically heterogeneous ICU population, with the explicit assumption that treatment effects may be heterogeneous.

Much of the progress that has been made in intensive care medicine is the result of identifying and abandoning potentially harmful interventions and treatments. Perhaps it is now time to add the indiscriminate use of fluid therapy to that list. Whenever tempted to give a fluid bolus to a patient, clinicians should remember “CIT TAIT” and should consider to just “sit tight”.
